# Allopregnanolone and mood in the peripartum: a longitudinal assessment in healthy women

**DOI:** 10.3389/fnbeh.2024.1499416

**Published:** 2024-11-21

**Authors:** Maria Katharina Grötsch, Ulrike Ehlert

**Affiliations:** Clinical Psychology and Psychotherapy, University of Zurich, Zürich, Switzerland

**Keywords:** allopregnanolone, neurosteroid, reproductive mood disorder, pregnancy, peripartum

## Abstract

**Background:**

Allopregnanolone (ALLO), a neuroactive steroid hormone derived from progesterone, can modulate mood via the GABA-A receptor. Peripartum mood can be influenced by psychosocial factors, previous mental illness, and hormonal changes. Studies suggest a U-shaped effect of ALLO on mood, with some women being more sensitive to hormonal changes than others. However, research in the peripartum is inconclusive.

**Methods:**

This study explored the link between salivary ALLO and mood during the peripartum. Over 12 weeks, N = 61 women completed the Edinburgh Postnatal Depression Scale and the State Anxiety subscale from the State–Trait Anxiety Inventory and provided saliva samples. Salivary ALLO was analyzed using an enzyme-linked immunosorbent assay, validated for saliva samples. Group-based trajectory modeling was performed to identify trajectories of ALLO courses. Multinomial logistic regression models were employed to identify risk factors associated with these trajectories.

**Results:**

ALLO levels increased during pregnancy and dropped 2 weeks before delivery. Three different trajectory groups of ALLO courses emerged (high decreasing, low moderate, low reduced). Trajectory groups were associated with distinct psychological risk factors, including previous mental illness, adverse childhood experiences, sleep problems, premenstrual symptoms, and resilience. The peripartum ALLO course showed a negative linear association with anxiety symptoms and a U-shaped association with depressive symptoms.

**Discussion:**

The consideration of individual ALLO courses can predict the risk for peripartum mood symptoms, particularly among women with preexisting risk factors. While the majority of women remain healthy during the peripartum transition, analyzing ALLO subgroups helps to provide a better understanding of the relationship between ALLO and peripartum mood.

## 1 Introduction

Allopregnanolone (ALLO), a neuroactive steroid hormone modulating gamma-aminobutyric acid (GABA-A) receptors, exerts manifold physiological and psychological effects within the female body (Reddy, 2010). The term “neuroactive steroid” traces back to Paul and Purdy (1992) and describes a hormone that can “*rapidly alter the excitability of neurons by binding to membrane-bound receptors*” (Paul and Purdy, 1992). During pregnancy, ALLO levels increase due to rising progesterone metabolism (Tsutsui and Haraguchi, 2021) and play an important role in physiological and psychological adaptations to pregnancy and fetal neurological development (Meltzer-Brody and Kanes, 2020; Schumacher et al., 2020). ALLO mainly exerts its neuroactive effect by binding to GABA-A receptors, its most studied effect, and is thus a positive allosteric modulator (Reddy, 2010). While it may have antidepressant, anxiolytic, and stress-relieving effects (Meltzer-Brody and Kanes, 2020), it is also associated with irritation or aggression (Andréen et al., 2009). During the peripartum, it is involved in the etiology of mood fluctuations (Andréen et al., 2009; Meltzer-Brody et al., 2018; Bäckström et al., 2011). Although the etiology of depressive and anxious mood symptoms during the peripartum period remains unclear, in 2019, ALLO was approved by the US Food and Drug Administration (FDA) as the first specific drug treatment for postpartum depression (Meltzer-Brody et al., 2018; Scott, 2019).

The complexity of peripartum mood fluctuations is further compounded by a multitude of interrelated factors such as psychosocial factors, past mental illness, and hormonal changes. In particular, a history of depression and anxiety (Al-Abri et al., 2023) as well as adverse childhood experiences (Guintivano et al., 2018) have been found to be predictors of peripartum depression. These factors can be exacerbated by sleep disturbances or a lack of social support (Al-Abri et al., 2023). Moreover, in line with the “sensitivity hypothesis” (Bloch et al., 2000), some women seem to be especially sensitive to hormonal fluctuations during reproductive transition phases, and react with mood symptoms, while others stay relatively stable, with studies suggesting a particular vulnerability to these hormonal changes in women with premenstrual symptoms or a history of reproductive depression (Bloch et al., 2000; O'Hara et al., 1991; Schiller et al., 2015; Liu et al., 2022).

Previous studies suggest that neurosteroids like ALLO may impact mood in a complex, dose-dependent manner. Consequently, ALLO is hypothesized to exert a U-shaped effect on mood regulation. Both insufficient and excessive ALLO secretions may contribute to mood disorders, while moderate levels optimize inhibitory neurotransmission and thus positive mood (Bäckström et al., 2011). This U-shaped effect is not restricted to ALLO but has also been observed with other GABA receptor ligands such as ethanol and benzodiazepines (Aguayo et al., 2002). According to the research, around 10 and 25% of individuals react with strong and moderate negative symptoms, respectively, to low doses of benzodiazepines, hormone replacements, or physiological hormone fluctuations (Weinbroum et al., 2001; Sundström et al., 1998; Andréen et al., 2006; Andréen et al., 2003). However, it remains unclear whether in sensitive women, negative mood symptoms occur only in response to low levels of ALLO or also in response to high levels, for example during pregnancy. Previous studies in women have mainly focused on investigating the sensitivity to relatively low ALLO levels, for example during the menstrual cycle or pregnancy (Andréen et al., 2003; Osborne et al., 2019; Hellgren et al., 2014). Studies demonstrating negative effects of high ALLO levels on mood are limited, and to our knowledge, there are no studies showing a full U-shaped effect. Thus, the hypothesis of a U-shaped effect of ALLO in peripartum women remains to be validated. Taken together, the sensitivity hypothesis and the U-shaped effect pose a methodological challenge, which warrants careful consideration when examining the relationship between ALLO and mood.

To date, studies investigating the relationship between ALLO and mood during the peripartum period have yielded conflicting findings. For instance, lower blood plasma ALLO levels during the second trimester were associated with higher anxiety symptoms postpartum (Osborne et al., 2019), and similarly, pregnant women with low blood plasma ALLO levels exhibited a stronger negative response to stress in the second trimester (Crowley et al., 2016). Moreover, lower ALLO levels have also been linked to more depressive symptoms in the third trimester of pregnancy and to “postpartum blues” (Hellgren et al., 2014; Nappi et al., 2001). However, some studies reported an association of higher ALLO levels with more anxiety but not depressive symptoms in at-risk women (Deligiannidis et al., 2019; Deligiannidis et al., 2016), and others found no significant correlation between ALLO levels and mood during the peripartum period at all (Epperson et al., 2006; Etyemez et al., 2023; Hellgren et al., 2017; Paoletti et al., 2006; Pearson Murphy et al., 2001; Wenzel et al., 2021). Overall, these mixed findings suggest that the complexity of the interaction between ALLO and mood might be better elucidated using a longitudinal approach, as cross-sectional studies can capture neither the U-shaped effect of ALLO on mood nor the inter-individual sensitivities to changes over time. Therefore, a simultaneous measurement of ALLO levels and mood may help to close this research gap.

For a reliable, longitudinal assessment of hormone fluctuations, saliva sampling constitutes an innovative, novel approach to measure ALLO levels. It is non-invasive, therefore stress-free and quick, making it particularly beneficial for vulnerable populations such as pregnant women (Hoyt and Granger, 2020). As participants can self-sample at home following instruction (Granger and Taylor, 2020), it has several advantages over the traditional measurement of ALLO in blood samples. In a recent study, we validated the measurement of ALLO in saliva with pregnant women in all three trimesters and found that an enzyme-linked immunosorbent assay (ELISA) kit, developed for analysis with blood samples, also reliably measured ALLO in saliva (Grötsch et al., 2022). The ELISA was tested regarding five parameters for optimal quality control for the quantification of ALLO. Samples were simultaneously analyzed with LC–MS, but the ELISA showed a more favorable assay range and sensitivity. The validation showed no matrix effects in the saliva that could interfere with the measurement. We now use this method to generate the first longitudinal hormone profile for salivary ALLO in the peripartum period.

In general, ALLO levels increase during pregnancy and drop immediately after birth (Etyemez et al., 2023; Pearson Murphy et al., 2001). However, the timing, increase, and drop of ALLO seem to vary inter-individually. Analyzing ALLO levels across individuals may therefore obscure the relationship between ALLO and mood because individual courses are not considered, which might explain the previous inconclusive results on the relationship between ALLO and mood in the peripartum. Accordingly, by analyzing individual ALLO courses in the peripartum period, it may be possible to identify sensitive subgroups (Gordon et al., 2021; Tepper et al., 2012; Eisenlohr-Moul et al., 2020) and to detect a potential U-shaped association with mood. While the sensitivity hypothesis suggests that some women may be more affected by ALLO changes than others (Bloch et al., 2000), the different effects of ALLO on mood in subgroups of women in the peripartum remain unclear.

Therefore, to improve the understanding of the relationship between ALLO and mood in the peripartum period, in the present study, a sample of healthy pregnant women provided weekly saliva samples over a period of 12 weeks to determine the physiological course of ALLO, and simultaneously completed weekly questionnaires to monitor mood changes. Studying healthy women can clarify the baseline role of endogenous ALLO in mood regulation under normal physiological conditions, free from the confounding effects of diseases, medication, or hormonal imbalances. As such, this approach could help to establish how mood is associated with naturally fluctuating ALLO in the peripartum period. Inter-individual differences in the course of ALLO were considered, using group-based trajectory modelling with the aim to identify different subgroups regarding ALLO courses. Further analyses were conducted to determine whether these subgroups can be predicted by mood symptoms in the peripartum period, which may shed light on whether there are women in the peripartum period who are exclusively sensitive to low or high ALLO levels or whether a U-shaped correlation may explain this relationship.

## 2 Materials and methods

The study was an observational, single-center, longitudinal study conducted at the University of Zurich, Switzerland. The data were collected between April 2022 and March 2023, with a mean study duration of 12 weeks per participant. Participants were compensated with a gift bag worth 100 CHF (approximately 112 US dollars). The study was evaluated by the cantonal ethics committee of the Canton of Zurich (KEK Zürich, Zürich, Switzerland) and classified as uncritical (BASEC Nr 2022–00220). Participants provided written informed consent. The study was preregistered on the Open Science Framework^1^.

### 2.1 Participants

**Figure 1:**
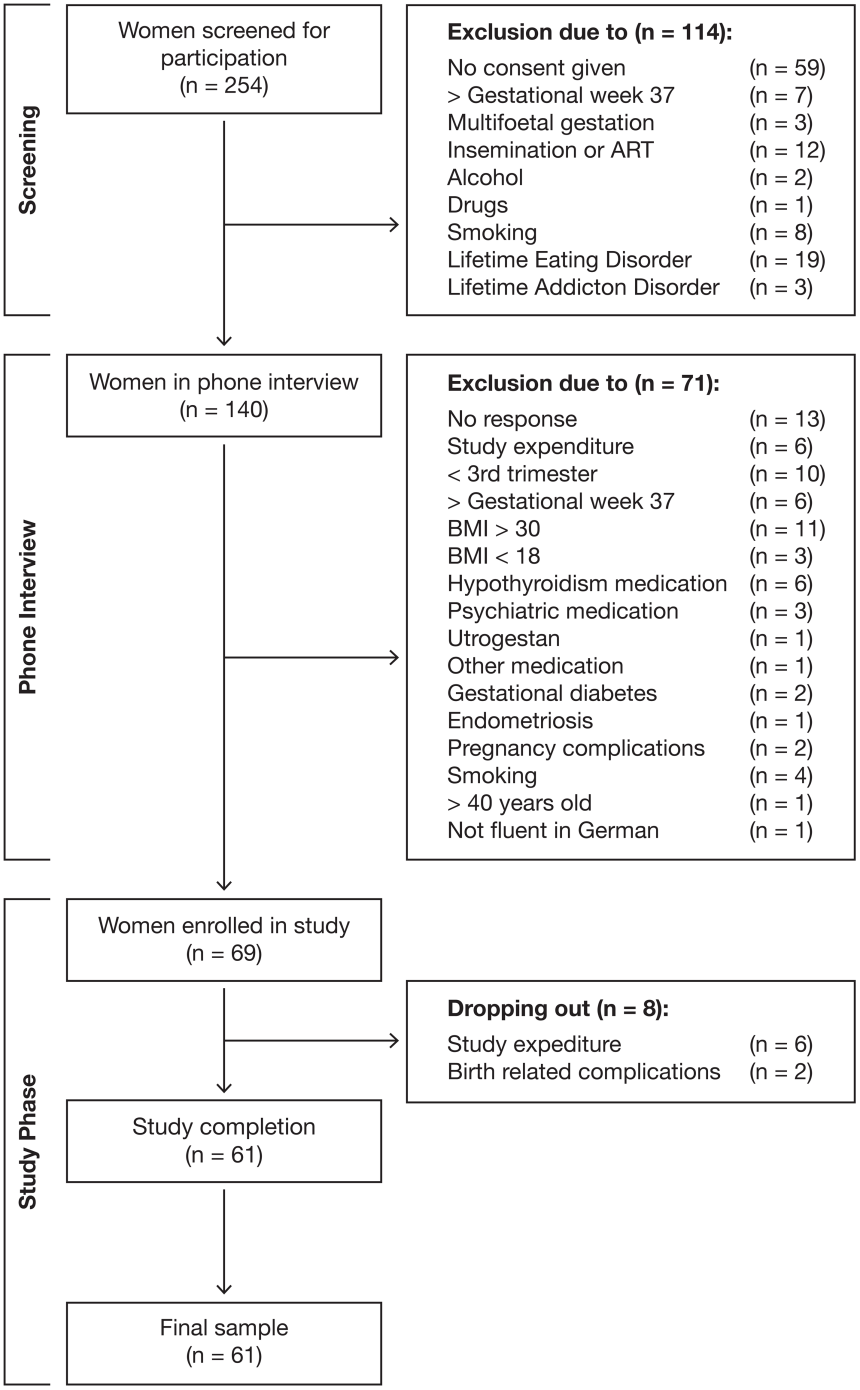
Flow of participants.

The participants were healthy pregnant women, aged between 20 and 40 years, in their third trimester. All participants provided written informed consent prior to participation in the study. Self-reported and double-checked eligibility criteria included questions about pregnancy and physical and mental health to exclude possible cofounders of the outcome variables. The following exclusion criteria were applied: multifetal gestation, artificial reproductive technologies and insemination, medical complications, medical conditions affecting ovarian function, current or history of psychosis, bipolar disorder, post-traumatic stress disorder, eating disorders, substance abuse or dependence, current medication use, drug use, smoking or alcohol consumption, and pre-pregnancy BMI >30 or < 18. Participants were recruited online through the study’s Instagram account, via paid advertisements, the department’s website, and through flyers in doctors’ offices, maternity ward waiting rooms, prenatal yoga classes, and birth centers. The final sample consisted of 61 women. The flow of participants is shown in Figure 1.

### 2.2 Study procedure

Interested women were screened using an online questionnaire. If eligible for study participation, they underwent a telephone interview, during which a trained clinical psychologist informed them about the study process, reviewed the eligibility criteria, and conducted a structured clinical interview for DSM-5 disorders. If all eligibility criteria were met, participants were enrolled in the study and an appointment was made for the first laboratory assessment between gestational weeks 34 and 36.

During the first laboratory assessment, the participants provided written informed consent. Participants were instructed on saliva collection and completed demographic and psychological questionnaires.

**Figure 2:**
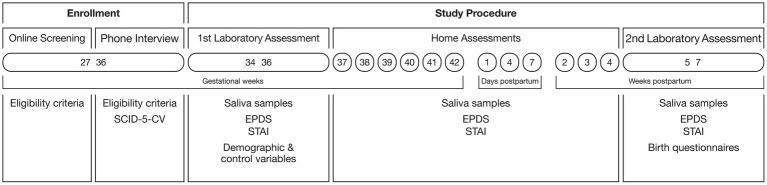
Study procedure.

Participants collected saliva and completed online questionnaires [Edinburgh Postnatal Depression Scale (EPDS), State–Trait Anxiety Inventory (STAI)] weekly from gestational week 37 up until birth, on days 1, 4, and 7 after birth (the day of the birth was day 0), and at 2, 3, and 4 weeks postpartum. The second laboratory assessment took place between 5 and 7 weeks postpartum. During this second visit, the participants handed over the frozen saliva samples, provided the final sample, and completed more psychological and birth-related questionnaires. An overview of the study procedure is provided in Figure 2.

### 2.3 Measures

We analyzed two main outcomes: ALLO hormone concentrations in saliva samples and psychological measures. Depressive and anxiety symptoms were collected longitudinally, while all other psychological questionnaires were completed at the first laboratory assessment.

#### 2.3.1 Saliva samples

Saliva samples were collected via passive drool using SaliCaps (IBL International GMBH, Hamburg, Germany). At each time point, participants filled three SaliCaps and provided 4.5–6 ml of unstimulated whole saliva. Participants were asked to refrain from eating, drinking, and brushing their teeth for 1 h prior to sample collection. Samples were immediately frozen and stored in participants’ home freezers, and were then brought to the second appointment in a cool box. Following the second laboratory assessment, samples were stored at −80°C until analysis at the University of Zurich. After thawing, samples were visually screened for contamination and excluded in the case of visible blood stains or food contamination. Storage times varied between 3 and 10 months. Analyses were conducted by a trained biologist at our laboratory.

##### 2.3.1.1 Allopregnanolone

ALLO was measured using an ELISA (Assay Genie (Ireland), SKU: UNEB0081), according to the manufacturer’s technical manual, using a 1:5 dilution of samples. The kit has an assay range of 31.2–2,000 pg/ml and a sensitivity of <9.5 pg/ml. In a previous study, we validated the kit for the measurement of salivary ALLO in pregnant women (Grötsch et al., 2022). The optical density of the plates was determined using a Tecan Infinite Plex. The concentrations were calculated using the software MagellanTM (TECAN, Version 7.3, Switzerland).

#### 2.3.2 Psychological measures

Psychological measures were assessed using validated German versions of online self-report questionnaires. For data collection, we used the Enterprise Feedback Suite (Tivian XI GmbH, 2021), a BSI-certified datacentre (ISO: 27001) compliant with the European General Data Protection Regulation (GDPR).

##### 2.3.2.1 Depressive symptoms

Depressive symptoms were assessed using the German version of the EPDS (Bergant et al., 1998), a validated screening method for peripartum depression. The scale consists of 10 self-report items rated on a 4-point Likert scale. The total score ranges from 0 to 30, with a cut-off of 10 indicating clinically relevant depressive symptoms.

##### 2.3.2.2 State anxiety symptoms

State anxiety symptoms were assessed using the State Anxiety subscale from the German version of the STAI (Grimm, 2009). The scale comprises 10 self-report items rated on a 4-point Likert scale (1–4), with a total score ranging from 10 to 40. The raw test values are converted into % agreement, ranging from 0 to 100.

##### 2.3.2.3 Adverse childhood experiences

Adverse childhood experiences were assessed using the subscales “emotional abuse from parents,” “physical abuse from parents,” “sexual abuse,” and “emotional neglect” from the German questionnaire on adverse childhood experiences (KERF-20; Isele et al., 2014). The scale consists of 20 self-report items, rated “yes” or “no,” and for different people (i.e., parents, siblings, peers, partners). Sum scores are calculated with the total sum of adverse experiences.

##### 2.3.2.4 Past anxiety disorder/major depression

Past anxiety disorder and major depression were assessed using the Structured Clinical Interview for DSM-5 Disorders—Clinician Version (SCID-5-CV; First et al., 2016). A past anxiety disorder included panic disorders, agoraphobia, social phobia, and generalized anxiety disorder but excluded specific phobias or separation anxiety. Past major depression included one or more episodes of major depression but excluded dysthymia alone. Women with affective disorders with psychotic or manic episodes were excluded from the study (see exclusion criteria).

##### 2.3.2.5 Birth anxiety

Birth anxiety was assessed using the German version of the Birth Anxiety Scale (GAS; Lukesch, 1983), which assesses anxiety around different birth situations in general. The scale comprises 25 self-report items rated on a 4-point Likert scale (0–4), with a total score from 0 to 75.

##### 2.3.2.6 Sleep problems

Sleep problems were assessed using the German version of the Pittsburgh Sleep Quality Index (PSQI; Riemann and Backhaus, 1996). The scale consists of 19 self-report items assessing subjective sleep quality within the last month, which generate seven “component” scores. The component scores are added to form a total sum score ranging from 0 to 21, with higher scores indicating more sleep problems.

##### 2.3.2.7 Premenstrual symptoms

Premenstrual symptoms before pregnancy were assessed using the German Premenstrual Syndrome (PMS) Inventory (Ditzen et al., 2011). The scale consists of 30 self-report items assessing typical symptoms that arise before menstruation, rated on a 4-point Likert scale (0–3), with a total score from 0 to 90.

##### 2.3.2.8 Resilience

Resilience was assessed using the Resilience Scale 11 (RS-11; Schumacher, 2004). The scale assesses resilience in general and consists of 11 self-report items rated on a 7-point Likert scale (1–7), with a total score from 11 to 77.

### 2.4 Statistical analysis

Missing data were addressed using the predictive mean matching method in the ‘mice’ package in R, generating 50 datasets to account for the uncertainty introduced by imputing missing values. This method imputes missing data based on the averages of similar observed data points, aiming to preserve statistical properties (Azur et al., 2011). It is particularly useful for handling variables with non-linear relationships, if the normality assumption is untenable, and for longitudinal data (White et al., 2011). Due to missed sample collection or samples outside the detection range, 34.6% of ALLO measurements and 7.0% of EPDS and STAI measurements were missing. After imputation, all participants and time points were used for analysis. Salivary ALLO and psychological measures were collected at the same gestational age (between gestational week 34 and 7 weeks postpartum). Subsequently, the time variable was centered around the individual delivery date to account for interindividual differences in gestational length. Time points before birth were coded negatively and time points after birth were coded positively, with the delivery date being 0. All models were calculated with this time variable. Group-based trajectory modelling (GBTM) was used to identify subgroups of ALLO profiles using the ‘gbmt’ package (Magrini, 2022). GBTM is a subtype of latent class growth models used to determine groups based on multivariate time series, in which people in the same group have a similar trajectory. The relationship between ALLO and psychological measures was analyzed using linear mixed-effects models in the whole sample and multinomial logistic regression (MLR) models to account for the trajectory groups. Model assumptions were checked using residual plots. The statistical threshold for significance was set at *p* = 0.05. Effect sizes are reported as *R^2^, η^2^,* and AOR. An *a priori* sample size estimation was performed using GPower (Faul et al., 2009), considering a medium effect size of *f^2^* = 0.3 with *α* = 0.05 and power = 0.95 (1 − ß), resulting in an estimated minimum sample size of *N* = 55 women. Statistical analyses were performed using RStudio version 2023.06.0–421. According to the model fit indices, presented in Supplementary Table S1, the data fit best with a model with three or four groups. Considering theoretical and clinical implications and the sample size, a model fit of three groups was chosen.

## 3 Results

### 3.1 Sample characteristics

Characteristics of participants are presented in Table 1. Participants were healthy pregnant women with an uncomplicated singleton pregnancy. The majority were Swiss and well-educated, 62% were married, and all but one were in a relationship with the child’s father. All women, of whom 57% were primiparous, gave birth to a term baby (*n* = 26/42.62% female assigned at birth). Mean gestational age at birth was *M* = 40.41 weeks (*SD* = 1.2, *MIN* = 37, *MAX* = 43). Forty-five women (73.8%) had a vaginal delivery, including four assisted vaginal deliveries (forceps/vacuum device). A further six (9.8%) had an elective caesarean section and 10 (16.4%) had an emergency caesarean section. Sixteen women (26.23%) had previous major depression and 14 (22.95%) had a previous anxiety disorder.

**Table 1:** Sociodemographic and health-related characteristics and group differences of different trajectory groups of salivary allopregnanolone.

Variable	Trajectory groups of salivary allopregnanolone								
	Total (*N* = 61)		Low moderate (*n* = 35)		Low reduced (*n* = 17)		High decreasing (*n* = 9)		*p, η^2^*
	*M*	*SD*	*M*	*SD*	*M*	*SD*	*M*	*SD*	
Age	32.0	4.3	31.8	4.21	31.7	4.42	33.4	4.08	n.s.
Pre-pregnancy BMI	22.5	2.8	22.2	2.94	22.7	2.47	23.5	2.23	n.s.
Gestational age at birth	40.5	1.3	40.6	1.17	40.2	1.19	40.8	1.15	n.s.
Mean depressive symptoms (EPDS)	5.3	4.5	5.27	0.53	5.98	0.98	5.74	1.18	<0.001^***^, 0.007
Mean state anxiety (STAI)	29.4	17.2	29.4	19.1	30.5	15.0	27.4	12.0	n.s.

*M*, mean; *SD*, standard deviation; *p*, significance level; n.s., not significant; EPDS, Edinburgh Postnatal Depression Scale; PSS, Perceived Stress Scale; STAI, State–Trait Anxiety Inventory; Affective symptoms averaged over the 14 time points in the peripartum; ^*^ < 0.05, ^**^ < 0.01, ^***^ < 0.001.

### 3.2 Trajectories of salivary allopregnanolone

Across all women, ALLO levels increased from the first measurement at 34 to 36 weeks of gestation and started to drop around 2 weeks prior to birth. However, there was a large inter-individual variance.

**Figure 3:**
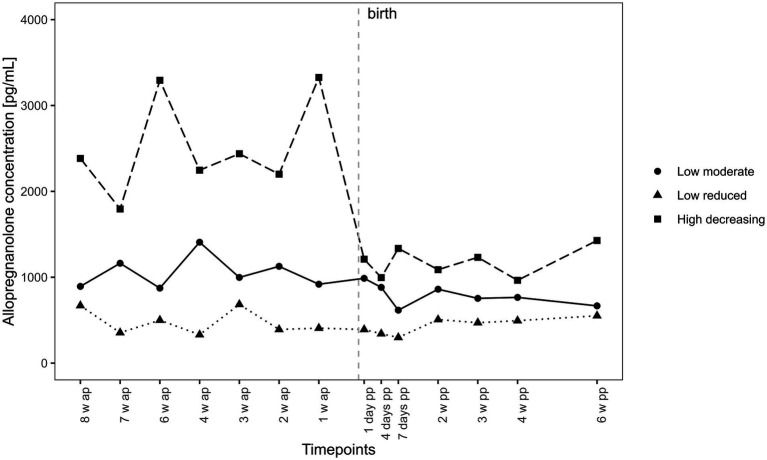
Trajectories of salivary allopregnanolone. Trajectories of salivary allopregnanolone over time. Salivary allopregnanolone was collected at the same gestational age (between gestational week 34 and 7 weeks postpartum). Subsequently, the time variable was centered around the individual delivery date to account for interindividual differences in gestational length. Time points before birth were coded negatively and time points after were coded positively with the delivery date being 0. All models were calculated with this time variable. Hence 8 weeks ante partum (w ap) is a different gestational week for each woman but 8 weeks before birth for each, whereas 2 weeks postpartum (pp) is the same for each woman.

As described above, three distinct groups in the trajectories of salivary ALLO emerged. These trajectories can be characterized by their intercept and slope. The detailed parameter estimates are listed in Supplementary Table S2. The three trajectories are shown in Figure 3.

The “high decreasing” group (*n* = 9, 14.7%) consists of women with the highest intercepts. These women exhibited high ALLO levels and high fluctuations during pregnancy, with a steep drop 1 week before birth. The “low moderate” group (*n* = 35, 57.3%) comprised the largest group. Women in this group started with a significantly lower intercept than the first group. The slope of this group is characterized by moderate fluctuations prepartum, with a moderate decrease within the first 7 days postpartum and relative stability between 2 and 6 weeks postpartum. The “low reduced” group (n = 17, 28.0%), making up almost a third of the sample, showed the lowest intercept and fluctuations, and unlike the other two groups, did not show a significant drop in ALLO levels around parturition.

### 3.3 Allopregnanolone and psychological measures

An overall mixed-effects model was calculated to analyze the relationship between ALLO and psychological measures in the whole sample. This method revealed a significant negative effect of time point (*β* = −0.106, *SE* = 0.047, *p* = 0.026^*^, *R^2^* = 2.6), confirming the observation that ALLO decreases over time. Moreover, a significant negative interaction between time point and anxiety symptoms (STAI) emerged (*β* = −0.003, *SE* = 0.001, *p* = 0.028^*^, *R^2^* = 2.1), suggesting that a decrease in ALLO results in an increase in anxiety symptoms. The overall model revealed no significant effects for depressive symptoms (EPDS).

An MLR was calculated to analyze the association between ALLO and psychological measures between the trajectory groups in the peripartum. Based on previous literature, nine psychological risk factors were selected for the model. When fitting the model, one category was chosen as the reference category and the others were compared to the reference. As over half of women fell into the “low-moderate” group, this was chosen as the reference for the regression models. The variables and results are presented in Table 2.

**Table 2:** Psychological factors associated with trajectory group membership of allopregnanolone.

	Low reduced		High decreasing	
Variable	AOR	95% CI	AOR	95% CI
Depressive symptoms (EPDS)	1.07^*^	1.00–1.15	1.13^*^	1.01–1.26
State anxiety (STAI)	0.98^*^	0.96–0.99	0.95^**^	0.92–0.98
Questionnaire on adverse childhood experiences (KERF-20)	1.11	0.99–1.22	1.29^***^	1.14–1.47
Previous anxiety disorder	2.81^**^	1.48–5.33	n.a.	n.a.
Previous major depression	0.31^***^	0.18–0.53	5.20^***^	2.68–11.01
Birth anxiety (GAS)	1.04^**^	1.01–1.06	0.85^***^	0.81–0.89
Sleep problems (PSQI)	0.92^*^	0.86–0.99	1.12^*^	1.02–1.24
Premenstrual symptoms (PMS)	0.99^*^	0.97–0.97	1.05^***^	1.03–1.07
Resilience (RS-11)	0.96^*^	0.93–0.99	0.82^***^	0.78–0.86

AOR, adjusted odds ratio; CI, confidence interval; EPDS, Edinburgh Postnatal Depression Scale; STAI, State–Trait Anxiety Inventory; KERF-20, Questionnaire on adverse childhood experiences; GAS, Birth Anxiety Scale—German Version; PSQI, Pittsburgh Sleep Quality Index; PMS, Premenstrual Syndrome Inventory; RS-11, Resilience Scale 11; ^*^ < 0.05, ^**^ < 0.01, ^***^ < 0.001.

Compared to the reference group (“low moderate”), women were more likely to be in the “low reduced” ALLO trajectory group if they had higher depressive symptoms and lower state anxiety. In contrast, they more frequently had a past anxiety disorder and reported more birth anxiety prepartum but were less likely to have past major depression. Additionally, they had fewer sleep problems prepartum and fewer premenstrual symptoms before pregnancy but had lower resilience.

Women were more likely to be in the “high decreasing” ALLO trajectory group if they had higher depressive symptoms and lower state anxiety compared to the reference group. They reported more adverse childhood experiences and were more likely to have past major depression. Moreover, they reported more sleep problems and more premenstrual symptoms prepartum but were less likely to report birth anxiety and had lower resilience.

The model showed an accuracy of 70.05% (*p* = <0.0001, *ϰ* = 0.4), with the highest sensitivity for the “low moderate” trajectory (94.70%) and the highest specificity for the “low reduced” trajectory (97.8%). The complete confusion matrix is shown in Supplementary Table S3.

**Figure 4:**
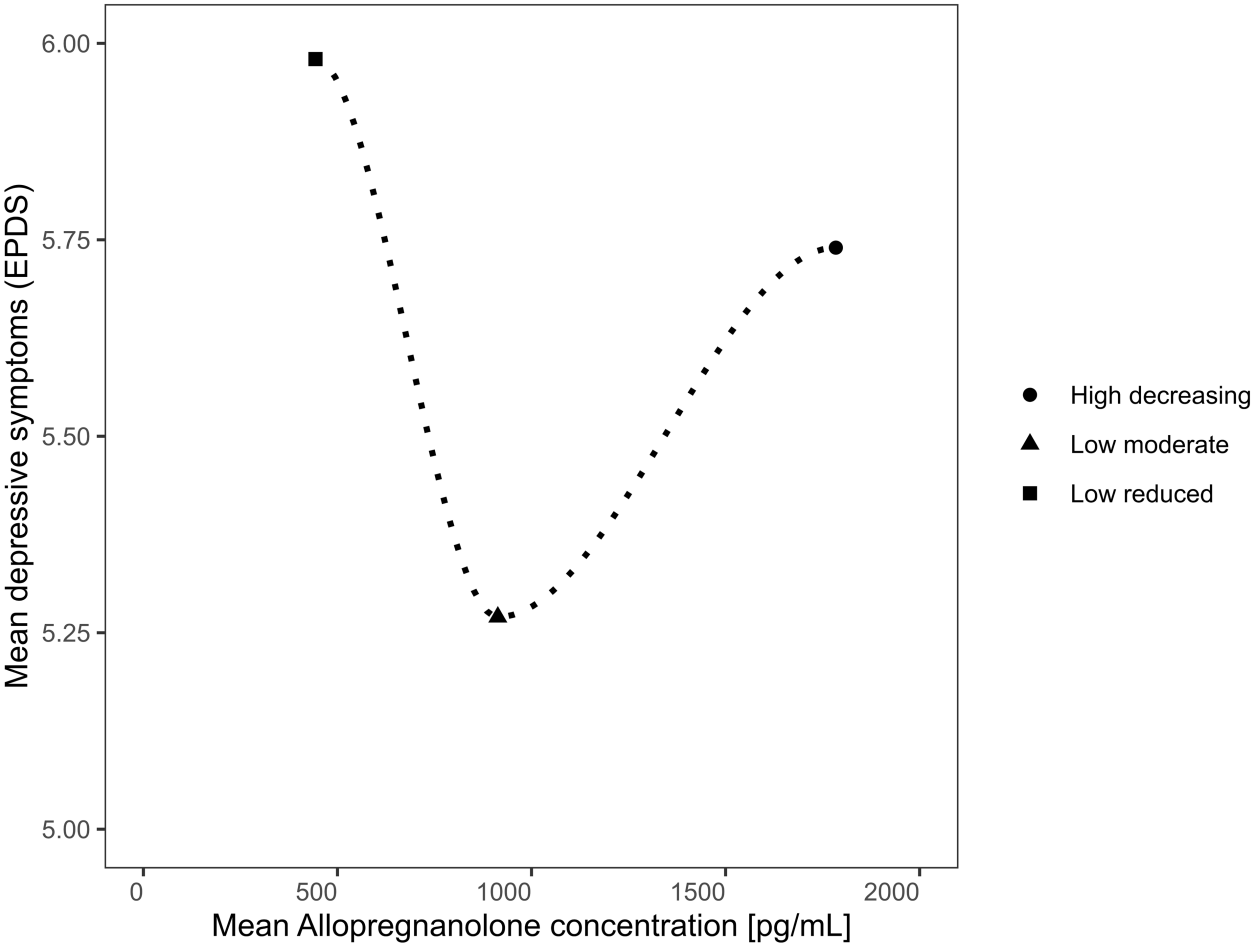
Relationship between depressive symptoms and allopregnanolone in the different trajectory groups.

The relationship between depressive symptoms and ALLO concentration across all subgroups is depicted in Figure 4. There was a significance difference in mean depressive symptoms between the three trajectory groups (small effect), as shown in the group comparisons in Table 1 and the MLR in Table 2. The relationship followed a U-shaped curve.

## 4 Discussion

The present study aimed to provide a comprehensive understanding of the relationship between ALLO and mood in the peripartum. In 61 healthy women analyzed over a 12-week peripartum period, we found that salivary ALLO levels increased in the third trimester of pregnancy and dropped after birth, but with a large inter-individual variance. The trajectory analysis resulted in three distinct groups. Over half of the women were characterized by relatively low levels of ALLO and moderate fluctuations during the peripartum, with a slight drop in ALLO levels shortly after birth. About a third of the women showed low ALLO levels and minor fluctuations over the course of the study, without a drop around parturition. The remaining women were characterized by relatively high levels and high fluctuations before birth. Women in this group already showed a steep decline in ALLO levels 1 week before parturition.

The three subgroups of women were associated with specific psychological profiles. Overall, ALLO levels were correlated with anxiety, confirming the findings of Osborne et al. (2019), but were not significantly associated with depressive symptoms, similar to previous findings (Osborne et al., 2019; Hellgren et al., 2017; Paoletti et al., 2006; Pearson Murphy et al., 2001; Wenzel et al., 2021). A history of major depression and anxiety disorder were separately associated with different ALLO courses in the peripartum. While various studies have analyzed ALLO in women with and without depressive or anxiety symptoms (e.g., Epperson et al., 2006; Etyemez et al., 2023; Pearson Murphy et al., 2001; Wenzel et al., 2021), previous research was unable to reliably establish a history of mood disorders as a predictor of ALLO levels in the peripartum period. One reason might be that subgroups are often formed using symptom scales rather than diagnostic interviews, leading to inaccurate estimates. Furthermore, defining subgroups based on symptoms instead of using ALLO levels may obscure the relationship, potentially leading to inconsistent results between studies.

The “sensitivity hypothesis” of Bäckström et al. (2011), as described in the introduction, has been previously confirmed for other GABA receptor ligands (Aguayo et al., 2002) and is now proposed for ALLO in healthy pregnant women in the peripartum period. In our study, ALLO showed a U-shaped association with depressive symptoms across all subgroups. This finding is supported by the results of a recent study investigating subgroups of depressive symptoms in relation to ALLO levels in the second trimester, which reported an association of persistent depressive symptoms with elevated ALLO levels (Björväng et al., 2024). Generally, higher ALLO levels may indicate a reduced GABA-A receptor sensitivity (Standeven et al., 2022; Maguire and Mody, 2008). This seems to be associated with a blunting of cortisol levels (Morrison et al., 2017), which in turn may account for more ALLO fluctuations. An explanation for this may be the negative feedback effect of ALLO on the HPA axis as a modulator of the stress response (Crowley et al., 2016; Morrison et al., 2017; Almeida et al., 2021). The present findings support this proposed mechanism, since women with higher ALLO fluctuations reported more adverse childhood experiences (see Table 2). Accordingly, an altered stress response, i.e., a blunting of the HPA axis hormones (Morrison et al., 2017; Hantsoo et al., 2023), might lead to more ALLO fluctuations during the peripartum, and an ALLO-mediated change in the stress response might explain an increased risk of depressive symptoms. Lower ALLO levels were also shown to be associated with depressive symptoms, representing the other end of the U-shaped ALLO level distribution (Hellgren et al., 2014; Nappi et al., 2001), and have been found to be associated with increased activity of the amygdala and insula and reduced activity in the dorsal medial prefrontal cortex (Sripada et al., 2013). Such brain activity appears to be linked to negative emotion and elevated anxiety (Sripada et al., 2013; Stein et al., 2007; Etkin and Wager, 2007). In our study, the group of participants showing the lowest ALLO levels reported higher birth anxiety. Elevated anxiety has been linked to increased HPA activation (Tafet and Nemeroff, 2020; Juruena et al., 2020), which might explain the reduced ALLO fluctuations due to the negative feedback in the stress response (Almeida et al., 2021).

Strengths of the study include the unique longitudinal design with repeated, validated, non-invasive salivary ALLO measurements and psychological assessments over 12 weeks. This is the first study to examine the relationship between ALLO and mood in saliva. Furthermore, trajectory analyses were used to discriminate subgroups of women according to ALLO and its association with psychological measures. The sample size was adequately assessed *a priori* and is in line with current longitudinal ALLO research on mood, with an average of *n* = 55 women (Grötsch and Ehlert, 2024). Limitations include the restricted generalizability due to the sample of physically and psychologically healthy, mostly well-educated Swiss women. While the findings suggest a potential relationship between ALLO and the identified subgroups, these findings should be viewed as preliminary and warrant further investigation in larger more diverse samples. Depressive symptoms are limited to mood fluctuations in an otherwise psychologically healthy group of women. As some ALLO levels were missing due to the samples being outside the detection range, the risk of overestimating had to be considered. However, due to the frequency of measurement, and the imputation using predictive mean matching, a well-established statistical technique (Azur et al., 2011) the risk of bias has been minimized. While the ELISA kit used was previously validated in saliva (Grötsch et al., 2022) and tested for specificity by the manufacturer, it is important to acknowledge that, as with many commercially available ELISA kits, there remains a potential for cross-reactivity with structurally similar hormones. This may affect absolute quantification to a limited extent (European Medicines Agency, 2011; Andreasson et al., 2015). In the present study, we focused on ALLO trajectories without considering symptom timing, whereas Björväng et al. (2024) focused on depressive symptom trajectories with only one ALLO measurement during the peripartum. Future studies could explore grouping women by ALLO levels and symptom development together in order to better understand their relationship during pregnancy and postpartum. Furthermore, as salivary ALLO measurement is still relatively new, it should be pitted against blood measurement in future studies.

In conclusion, the present study contributes to our understanding of inter-individual differences in ALLO courses and their relationship with mood. Using longitudinal analysis, three distinct subgroups were identified, characterized by different ALLO levels and fluctuation patterns. These subgroups each showed a unique psychological profile, possibly supporting the “sensitivity hypothesis” (Bloch et al., 2000). The results reveal that in the peripartum period, both low and high ALLO levels influence depressive symptoms in a U-shaped manner. ALLO levels were negatively associated with anxiety symptoms. A history of mood disorder, adverse childhood experiences, premenstrual symptoms, birth anxiety, and sleep problems on the one hand, as well as resilience on the other hand, were associated with the course of ALLO in the peripartum. Overall, the results highlight the importance of considering subgroups of women during the peripartum in order to promote the understanding of and consequently find appropriate treatment for mood disorders.

## Data Availability

The raw data supporting the conclusions of this article will be made available by the authors, without undue reservation.

## Footnotes

^1^ https://osf.io/
