# Supplementary material for: Allopregnanolone and mood in the peripartum: a longitudinal assessment in healthy women
Source: Front Behav Neurosci. 2024 Nov 21;18:1499416. doi: 10.3389/fnbeh.2024.1499416 (PMC11617183; doi:10.3389/fnbeh.2024.1499416)
Supplement: Supplementary file 1 [file Table_1.DOCX]

Supplementary Material

# Supplementary Tables

**Table S1.** Information criteria for group-based trajectory models of salivary allopregnanolone

| **Groups** | **Parameter** | **AIC** | **BIC** | **Log Likelihood** |
| --- | --- | --- | --- | --- |
| 1 | 3 | 12372.62 | 12386.47 | -6183.31 |
| 2 | 6 | 12169.16 | 12196.86 | -6078.58 |
| 3 | 12 | 12097.03 | **12152.44** | -6036.51 |
| 4 | 16 | **12088.14** | 12162.01 | -6028.07 |
| 5 | 21 | 12098.59 | 12195.56 | -6028.30 |

**Note**. AIC = Akaike’s information criterion; BIC = Bayesian information criterion.

**Table S2.** Parameter estimates for allopregnanolone trajectory

| **Variable** | **Trajectory groups of salivary allopregnanolone** | | |
| --- | --- | --- | --- |
|  | **Low**  **moderate**  (n = 35) | **Low**  **reduced**  (n = 17) | **High**  **decreasing**  (n = 9) |
| Intercept | 1222.46*** | 562.78*** | 2918.04*** |
| Slope | -40.24*** | -40.24*  2.58 | -146.47*** |

**Note.** * <0.05, ** <0.01, ***<0.001.

**Table S3.** Confusion matrix of the multinomial logistic regression model predicting trajectory groups of allopregnanolone with psychological factors

| **Variable (%)** | **Trajectory groups of salivary allopregnanolone** | | |
| --- | --- | --- | --- |
|  | **Low**  **moderate**  (n = 35) | **Low**  **reduced**  (n = 17) | **High**  **decreasing**  (n = 9) |
| Sensitivity | 94.70 | 25.62 | 54.96 |
| Specificity | 41.72 | 97.80 | 95.45 |
| Positive predictive value | 69.19 | 81.25 | 67.78 |
| Negative predictive value | 85.06 | 77.92 | 92.40 |
| Prevalence | 58.02 | 27.14 | 14.84 |
| Detection rate | 54.95 | 6.95 | 8.16 |
| Detection prevalence | 79.41 | 8.56 | 12.03 |
| Balanced Accuracy | 68.21 | 61.71 | 75.20 |
